# Different Blood Metabolomics Profiles in Infants Consuming a Meat- or Dairy-Based Complementary Diet

**DOI:** 10.3390/nu13020388

**Published:** 2021-01-27

**Authors:** Minghua Tang, Nicholas E. Weaver, Lillian M. Berman, Laura D. Brown, Audrey E. Hendricks, Nancy F. Krebs

**Affiliations:** 1 Section of Nutrition, Department of Pediatrics, School of Medicine, University of Colorado Anschutz Medical Campus, Aurora, CO 80045, USA; lillian.berman@cuanschutz.edu (L.M.B.); Nancy.Krebs@cuanschutz.edu (N.F.K.); 2 Department of Mathematical and Statistical Sciences, University of Colorado Denver, Denver, CO 80204, USA; NICHOLAS.WEAVER@UCDENVER.EDU (N.E.W.); Audrey.Hendricks@ucdenver.edu (A.E.H.); 3 Section of Neonatology, Department of Pediatrics, School of Medicine, University of Colorado Anschutz Medical Campus, Aurora, CO 80045, USA; laura.brown@cuanschutz.edu

**Keywords:** infant nutrition, protein-rich foods, growth, metabolomics

## Abstract

Background: Research is limited in evaluating the mechanisms responsible for infant growth in response to different protein-rich foods; Methods: Targeted and untargeted metabolomics analysis were conducted on serum samples collected from an infant controlled-feeding trial that participants consumed a meat- vs. dairy-based complementary diet from 5 to 12 months of age, and followed up at 24 months. Results: Isoleucine, valine, phenylalanine increased and threonine decreased over time among all participants; Although none of the individual essential amino acids had a significant impact on changes in growth Z scores from 5 to 12 months, principal component heavily weighted by BCAAs (leucine, isoleucine, valine) and phenylalanine had a positive association with changes in length-for-age Z score from 5 to 12 months. Concentrations of acylcarnitine-C4, acylcarnitine-C5 and acylcarnitine-C5:1 significantly increased over time with the dietary intervention, but none of the acylcarnitines were associated with infant growth Z scores. Quantitative trimethylamine N-oxide increased in the meat group from 5 to 12 months; Conclusions: Our findings suggest that increasing total protein intake by providing protein-rich complementary foods was associated with increased concentrations of certain essential amino acids and short-chain acyl-carnitines. The sources of protein-rich foods (e.g., meat vs. dairy) did not appear to differentially impact serum metabolites, and comprehensive mechanistic investigations are needed to identify other contributors or mediators of the diet-induced infant growth trajectories.

## 1. Introduction

Evidence-based consensus holds that the first year of life is significant for obesity programming, and undesired growth patterns in infancy, namely excessive weight relative to length, are strongly associated with childhood obesity [1]. Nutrition plays a critical role in infant growth trajectories, and dietary protein has been a subject of interest because of its controversial role in infant growth. Some research showed that a high protein intake had been associated with increased overweight risks [2,3], while others showed no growth difference between high- and low protein intakes [4]. Most of these studies focused on the high-protein content of infant formula, and research is limited in the effect of protein source from complementary foods on infant growth, especially among different types of protein-rich foods. Complementary feeding, defined as the addition of solid or semi-solid foods added to infants’ diets, usually starts at 5–6 months of age [5]. Dietary diversity significantly increases during this time, including dietary protein source [6]. Research in adults and animal models has shown that types of protein-rich foods can differentially impact various health indicators, including insulin resistance [7], cancer risk [8], and bone health [9]. Research is limited, however, in evaluating different types of protein-rich foods during complementary feeding on infant growth and risk of obesity.

Our group recently completed a randomized controlled feeding study that compared two types of protein-rich foods (meat vs. dairy) as the main source of protein from complementary foods between 5 and 12 months of age on infant growth [10]. A follow-up assessment was conducted at 24 months. We discovered that length-for-age Z score (LAZ, linear growth parameter) increased in the meat group and decreased in the dairy group from 5 to 12 months, which resulted in a significant increase of overweight parameter weight-for-length Z score (WLZ) and increased risk of overweight in the dairy group [10]. These divergent growth patterns also persisted at 24 months, one year after the intervention ended [11]. However, mechanisms of the differential growth trajectories in response to dairy vs. meat protein remain unclear. Previous studies showed that a higher protein intake (e.g., infant formula compared to breastmilk) led to increased circulating IGF-1 and insulin [3], which exerted anabolic effects and accelerated weight gain in formula-fed infants. However, IGF-1 did not significantly differ between the two groups of infants in our study [10], when participants consumed the same quantities of protein but from different sources. Emerging research showed that circulating amino acids (e.g., branched-chain amino acids or BCAAs) and acyl-carnitine may also play a role in regulating infant growth and weight gain [12]. The objective of this study was to investigate the mechanisms responsible for different growth trajectories in infants consuming a meat- or dairy-based complementary diet. We used a targeted approach to quantitatively measure amino acid and acyl-carnitine concentrations and an untargeted approach to explore new pathways that might differ between the two study groups.

## 2. Materials and Methods

### 2.1. Study Design

Blood samples were collected from participants in a randomized controlled trial conducted in the metro Denver area in the United States [10]. In brief, full-term infants who were exclusively formula-fed were recruited and randomized to consume a meat- or dairy-based complementary diet from 5 to 12 months of life, with meat- or dairy as the primary source of protein of complementary foods. Total protein intake during the intervention was targeted at 15% of energy or 3 g/kg/day, which was higher than the participants’ reported habitual intake (~10% or 2.1 g/kg/d) at 5 months. The meat group consumed pureed beef, pork, and poultry (provided), and the dairy group consumed yogurt and cheese (provided). The same type of cow-milk-based formula was also provided to all participants by the research team. The amount of formula consumed between groups did not significantly differ during the intervention [10]. Both groups consumed comparable amounts of protein and minimal protein from the assigned alternative. Participants’ length and weight were assessed every month from baseline (5 months) to the end of the intervention (12 months). Venous blood samples were collected at 5 and 12 months.

One year after the intervention ended at 12 months of age, an observational follow-up visit was conducted at 24 months with length and weight measurements and blood collections. The purpose of the 24 months follow-up was to determine if there were lasting effects of the dietary intervention from 5–12 months. Blood samples were collected at 5, 12 and 24 months at Children’s Hospital Colorado by the Clinical and Translational Research Center pediatric nurses. Up to three attempts were taken to draw blood. If blood could not be obtained by the 3rd attempt, “no blood sample” was noted for the participant’s visit. This study was approved by the Colorado Multiple Institutional Review Board and was registered at ClinicalTrials.gov (NCT02142647).

### 2.2. Sample Analysis

Blood samples were centrifuged and serum was stored at −80 degrees C until analysis. Targeted and untargeted metabolomics analyses were conducted by the Metabolomics Core Lab at University of Colorado Anschutz Medical Campus. Serum samples were thawed on ice, then 20 µL of each was extracted with 480 µL of ice-cold lysis/extraction buffer (5:3:2 methanol:acetonitrile:water) containing 2.5 µM of stable isotope-labeled amino acid mix (Cambridge Isotope Laboratories, cat no MSK-A2-1.2) and a mix of acylcarnitines (Cambridge Isotope Laboratories, NSK-B-1) diluted 1 to 200 to serve as standards. Samples were extracted and analyzed on a Vanquish ultra-High Performance Liquid Chromatography Work (UHPLC) coupled online to a Q Exactive mass spectrometer (Thermo Scientific) using a high throughput 5 min C18 gradient in positive and negative ion modes (separate runs) [13]. All samples were analyzed in one batch. Metabolite assignments for relative and absolute quantification were performed using data analysis software Maven exactly as described [14,15]. For absolute quantification, circulating amino acids, acyl-carnitines and trimethylamine N-oxide (TMAO) concentrations were measured.

### 2.3. Statistical Approach

All statistical analyses were performed using R (version 3.5.1). For the intervention period (5 to 12 months), the mean change in growth Z-scores (i.e., length-for-age Z score (LAZ), weight-for-length Z score (WLZ), weight-for-age Z score (WAZ), head circumference-for-age Z score (HCAZ)) were regressed in a linear model on diet (dairy or meat protein), change in metabolite concentration, and the interaction between diet and change in metabolite concentration. A separate regression was performed for each of the four growth Z-scores and 32 quantified metabolites resulting in 128 models. This was repeated for the post intervention period (12 to 24 months) to assess whether differences persisted beyond intervention. Next, we assessed the effect of diet on change in metabolite concentrations by modeling change in each of the 32 metabolite concentrations as the outcome and diet as the predictor in a linear regression. Given previous research indicating a strong relationship between TMAO and meat intake, we assessed the relationship with TMAO and the intervention period (5 to 12 months) in the meat only group.

Principal components (PCs) were constructed from the change in standardized metabolite concentrations (12 months–5 months) for the eight essential amino acids identified in the targeted metabolomic assays. As with the individual metabolites, two types of linear regression models were used: (1) PC as the outcome and diet group as the primary predictor, (2) difference in growth Z-scores as the outcome and PCs as the predictors. For the second type of model, interaction between PC and diet group was also assessed. For the untargeted metabolomic compounds, regression analysis was performed as described above using a log-transformation of the change in the metabolite concentration. Benjamini-Yekutieli procedures were used to control the false discovery rate (FDR) at 0.05 within each set of hypothesis tests: targeted metabolites with diet, targeted metabolites with growth outcomes, untargeted metabolites with diet, untargeted metabolites with growth outcomes, PCs with diet, and PCs with growth outcomes.

## 3. Results

### 3.1. Subjects

**Figure 1:**
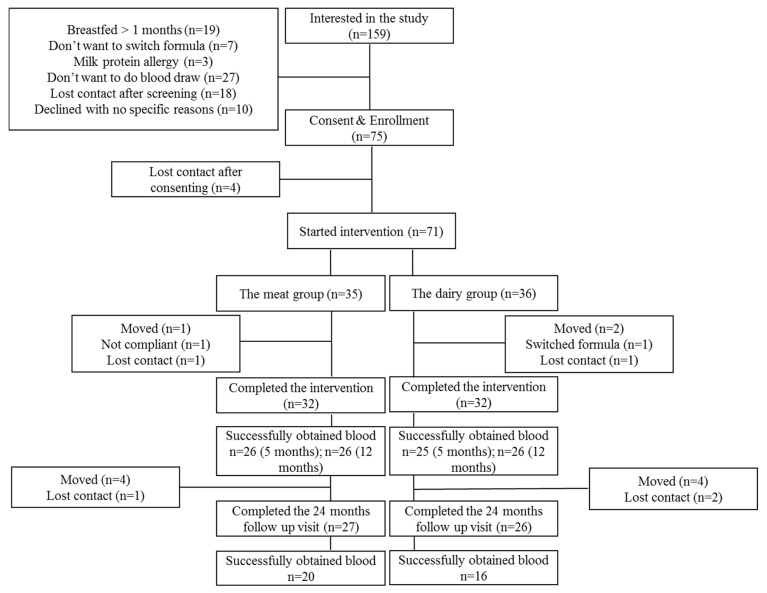
Consort Diagram.

A total of 64 infants completed the dietary intervention (*n* = 32 per group) with weight and length data from 5 to 12 months of age, and 53 infants completed the 24 months observational follow up (Meat group *n* = 27, Dairy group *n* = 26). Blood samples were successfully collected from 51 infants at 5 months (Meat group *n* = 26, Dairy group *n* = 25), 52 infants at 12 months (Meat group *n* = 26, Dairy group *n* = 26) and 36 infants at 24 months (Meat group *n* = 20, Dairy group *n* = 16) (Figure 1). Even though our overall follow-up rate at 24 months was 82%, the lower number of blood samples available for analysis at this time point was secondary to (1) unsuccessful phlebotomy after a maximum of three attempts or (2) parents/caregivers refused to allow for more than one attempt to draw blood. Table 1 summarizes growth Z scores of those participants from whom blood samples were successfully collected at 5, 12 and 24 months. There were no significant differences between groups for birth length, sex, maternal BMI or maternal education. Mothers were, on average, overweight as defined by BMI between 25 and 29.9. Demographics did not differ between non-participants and participants at 24 months [11]. Consistent with the report of growth Z scores in the parent cohort (*n* = 64) [10], during the intervention, there was a significant group-by-time interaction (*p* = 0.001) of length-for-age Z score (LAZ) from 5 to 12 months, indicating that LAZ increased in the Meat group compared to the dairy group. WAZ increased among all participants from 5 to 12 months. Changes in WAZ and LAZ led to a significant group-by-time interaction (*p* = 0.02) of WLZ. Although participants were off the dietary intervention at 12 months of age and started to consume comparable complementary diets between the meat and dairy groups, these growth trajectories persisted at 24 months (Table 1). These relationships are comparable to the parent cohort that was previously published [11].

### 3.2. Essential Amino Acids

Concentrations of some essential amino acids, including isoleucine (*P_FDR_* = 0.0467, P_raw_ = 0.0146), valine (*P_FDR_* = 0.0011, *P_raw_* = 0.0007), phenylalanine (*P_FDR_* = 0.0015, *P_raw_* = 0.0001) increased and threonine (*P_FDR_* = 0.0098, *P_raw_* = 0.0021) decreased from 5 to 12 months, without significant differences between diet groups (unadjusted *p*-values of 0.5122, 0.6393, 0.6848 and 0.1179, respectively; Table 2). There was no statistically significant effect from 12 to 24 months (Table 2).

None of the individual essential amino acids had a significant impact on changes in growth Z scores from 5 to 12 months of age. When using principal component analysis to further assess the relationship between the essential amino acids and growth Z scores, PC2 was heavily weighted by BCAAs (leucine, isoleucine, valine) and phenylalanine, and had a significant association with LAZ. This principal component had a positive association with changes in LAZ from 5 to 12 months (*P_FDR_* = 0.0815, *P_raw_* = 0.010) when controlling for diet group but not considering a PC vs. group interaction term. Inclusion of the interaction term resulted in a nominally significant PC2 vs. group interaction (*P_FDR_* = 0.3883, *P_raw_* = 0.0485) showing that the effect of PC2 was stronger in the meat group (0.346) compared with dairy (0.050). PC2 was not significantly associated with WAZ or WLZ changes regardless of the inclusion of an interaction term (*P_raw_* = 0.1286 and *P_raw_* = 0.7, respectively for the models without an interaction term).

### 3.3. Acylcarnitines & TMAO (Quantitative)

Concentrations of acylcarnitine-C4 ($P_{FDR}=0.0467$, $P_{raw}=0.0140$), acylcarnitine-C5 ($P_{FDR}=0.0136$, $P_{raw}=0.0034$), and acylcarnitine-C5:1 ($P_{FDR}=0.0040$, $P_{raw}=0.0005$) significantly increased from 5 to 12 months of age among all participants without statistically significant differences between groups (unadjusted *p*-values of 0.0999, 0.3541 and 0.6493, respectively. See Table 3 for full results). From 12 to 24 months of age, acylcarnitine-C4 decreased in both groups (*p* = 0.0045). None of the acylcarnitines were associated with infant growth Z scores. In the model containing both dairy and meat intervention groups, concentrations of TMAO were not significantly different between meat and dairy groups ($P_{FDR}=\;$0.7546) and did not change significantly over time from 5 to 12 months ($P_{FDR}=0.4147,\;P_{raw}=0.2851$) or from 12 to 24 months ($P_{FDR}=0.7800$, $P_{raw}=0.7750$) and were not significantly associated with infant growth Z scores (FDR adjusted *p*-values for LAZ, WAZ, WLZ, and HCAZ of 0.6168, 0.6251, 0.9920 and 0.5962, respectively). However, in the meat only stratified analysis, TMAO significantly increased from 5 to 12 months ($P_{FDR}=0.0414,\;P_{raw}=$ 0.0049).

### 3.4. Untargeted Metabolomics

Overall, 151 metabolites were annotated by the untargeted metabolomics analysis in both hydrophilic and hydrophobic modes. Consistent with the targeted analysis, threonine decreased ($P_{FDR}=0.0037$, $P_{raw}<0.0001$) and valine increased ($P_{FDR}=0.0205$, $P_{raw}=0.0002$) from 5 to 12 months without differences between groups (unadjusted *p*-values of 0.0594 and 0.8713, respectively). Acylcarnitine-C5 ($P_{FDR}=0.0205$, $P_{raw}=0.0003$) and acylcarnitine-C5:1 ($P_{FDR}=0.0012$, $P_{raw}<0.0001$) also increased from 5 to 12 months in both groups. There were no significant changes of the metabolites from 12 to 24 months. We also ran the association of gender and change in metabolite for both targeted and untargeted data. When we adjust for multiple tests, none of the metabolites passed a FDR *p* value of 0.05.

## 4. Discussion

This study aimed to identify metabolites in serum that are associated with consuming the meat- and dairy-based complementary diet and infant growth. We found that consuming a high-protein complementary diet (~15% of total energy) significantly increased serum concentrations of several essential amino acids, including phenylalanine, BCAA isoleucine, and valine from baseline (~10% of total energy from protein). These findings were expected because all participants increased their protein intake from 2.1 g/kg/d to 3.2 g/kg/d from 5 to 12 months of age [10]. One adult cohort showed that habitually consuming meat, fish and vegetarian-based diets led to significantly different concentrations of circulating essential amino acids, which were associated with dietary protein quality and quantity [16]. A number of intervention studies also reported that plasma essential amino acid concentrations depend upon dietary protein quality, with higher essential amino acid concentrations associated with animal protein intakes [17,18]. In our study, we did not observe significant differences in relative metabolite amounts between the meat and dairy groups. Because both meat and dairy foods are considered high-quality protein sources according to the WHO digestible indispensable amino acid score, consuming the same quantity likely contributed to comparable circulating amino acid profiles as shown in our study.

Insulin-like growth factor I (IGF-1) has been proposed to be the key mediator of WLZ difference found between groups consuming a high- vs. low-protein infant formula [19]. This concept is also called the “early protein hypothesis” [20]. In our study, infants from both the meat- and dairy-groups gained comparable amounts of weight, which were consistent with similar increases in IGF-1 concentrations in both groups [10]. Thus, differences in IGF-1 concentrations between meat and dairy groups did not explain the distinctive length gain in the meat group compared to dairy group (e.g., LAZ). We also ran the same models for the untargeted metabolomics data with change in IGF-1 value as the outcome, but none of the metabolites passes an FDR of 0.05. Although none of the individual amino acids were associated with growth Z scores of the participants, there was a nominally significant positive association between the BCAAs and phenylalanine principal component (PC2) with LAZ. This could suggest that protein-rich foods high in BCAAs and phenylalanine may promote infant linear growth. However, more research is needed to directly assess the potential impact of BCAAs on infant linear growth. Besides the potential effect of increasing insulin and IGF-1, BCAAs, especially leucine, have been considered potent nutrient signals to induce muscle synthesis [21] and bone density [22,23]. This may partially contribute to the association of BCAAs and LAZ in our study. Although a number of previous studies have reported that high quality, animal-based protein improves linear growth in infants and young children at risk of growth faltering from low-resource settings [24,25], it is still not clear how the sources of animal-based protein, such as meat and dairy, could differentially impact infant growth trajectories. Moreover, although dairy foods, especially whey protein, tend to have higher BCAA contents compared with meat [26], some research in overweight adults reported that meat intake was associated with higher BCAA concentrations while dairy was not [27]. It is also possible that constituents beyond protein in these protein-rich foods, such as vitamins and minerals, could potentially affect infant growth and weight gain, and that it is not protein but other compounds in protein-rich foods that could affect infant growth. For example, meat and dairy foods also had strikingly different iron contents. Because other diet-induced changes, such as gut microbiota, could be a potential mediator of dietary protein and infant growth [28] and iron is a key nutrient that could modulate developing gut microbiota [29]. These speculations clearly require further investigation. Nonetheless, these compounds in meat and dairy-rich foods are still integral parts of the whole foods, and findings are valid in terms of dietary patterns on infant growth and risk of overweight, especially when these dietary patterns during the first year of life had lasting effects.

We found an increase in short-chain acyl-carnitine concentrations (C4 and C5) from 5 to 12 months without group differences. Acyl-carnitines are fatty acid oxidation intermediates and short-chain acyl-carnitines can be produced as part of BCAA degrading products [30]. These findings were consistent with a previous study in 6-month-old infants showing that C4- and C5-acylcarnitines increased with high-protein formula consumption [3]. These short-chain acyl-carnitines have been found to associate with insulin resistance in adults [30], but the effects in infants are not clear. Short-chain acyl-carnitines are also emerging as a generic indicator of meat intake [31], although the concentrations increased in both meat and dairy groups in our study. We did not find any changes over time in long-chain acyl-carnitines, which are considered an indicator of fatty-acid oxidation and are reportedly higher in obese adults [32]. TMAO, a molecule generated from choline, betaine, and carnitine via gut microbial metabolism, is emerging as a potent biomarker for cardiovascular disease and strongly associated with meat intake [33]. However, most of the research on TMAO and meat intake has been conducted in adults. In our study, the meat group significantly increased protein intake from 5 to 12 months and the primary source of protein from complementary foods was meat; TMAO did not increase significantly from 5 to 12 months in the meat group, not dairy. However, TMAO concentrations dropped from 12 to 24 months as the intervention ended at 12 months. Thus, the long-term impact of meat intake on infant’s cardiovascular function and the biological significance of TMAO during infancy require further investigation.

There are several limitations of this study. First, we had a relatively small sample size and power calculation was based on the primary outcome infant growth. It is possible that we were underpowered for the metabolomics analysis. Second, only formula-fed infants were studied which reduced the generalizability of the findings. Third, we did not include a “reference” or “control” group without dietary protein intervention, although it is challenging to define a standard complementary diet since recommendations are generally lacking. 

## 5. Conclusions

Overall, our findings suggest that increasing total protein intake from ~2 g/kg/d at 5 months to 3.2 g/kg/d by providing protein-rich complementary foods was associated with increased concentrations of certain essential amino acids and short-chain acyl-carnitines. The sources of protein-rich foods (e.g., meat vs. dairy) did not appear to differentially impact serum metabolites. In addition, serum BCAAs may at least partially contribute to infant linear growth during complementary feeding. Future comprehensive mechanistic investigations are needed to identify other contributors or mediators of the diet-induced infant growth trajectories.

## Figures and Tables

**Table 1:** Subject characteristics ^1^.

	Meat Group	Dairy Group	*p*-Value
Birth weight (kg)	3.30 ± 0.41	3.32 ± 0.39	0.45
Maternal BMI ^2^	28 ± 6	28 ± 7	0.88
5 months LAZ	−0.19 ± 0.86	−0.30 ± 1.02	0.63
5 months WAZ	−0.03 ± 0.69	−0.14 ± 0.82	0.59
5 months WLZ	0.08 ± 0.79	0.04 ± 0.66	0.80
12 months LAZ	0.14 ± 0.90	−0.60 ± 0.91	0.002
12 months WAZ	0.40 ± 0.74	0.39 ± 0.78	0.99
12 months WLZ	0.95 ± 0.90	0.33 ± 0.48	0.04
24 months LAZ	0.19 ± 0.62	−0.37 ± 0.60	0.01
24 months WAZ	0.40 ± 0.56	0.31 ± 0.89	0.65
24 months WLZ	0.55 ± 0.99	0.34 ± 0.70	0.47

^1^ Mean ±SD, ^2^ Independent Student’s t test. LAZ: length-for-age Z score; WAZ: weight-for-age Z score; WLZ: weight-for-length Z score.

**Table 2:** Concentrations of serum essential amino acids (µM) at 5, 12 and 24 months between the meat and dairy groups ^1^.

							Meat Vs. Dairy ^2^ *p*-Value		Change over Time ^2^ *p*-Value	
Amino Acids	Meat 5 m	Dairy 5 m	Meat 12 m	Dairy 12 m	Meat 24 m	Dairy 24 m	12–5 m Model	24–12 m Model	12–5 m Model	24–12 m Model
L_Histidine	86.7 ± 21.3	76.5 ± 12.3	94.8 ± 17.02	89.4 ± 13.4	94.3 ± 13.1	81.7 ± 13.3	0.8596	0.6570	0.0500	0.0901
L_Leucine	83.2 ± 33.8	74.5 ± 24.2	93.4 ± 32.4	101.8 ± 47.5	66.2 ± 20.0	65.0 ± 20.8	0.8596	0.9979	0.1202	0.0819
L_Isoleucine	124.6 ± 44.1	113.8 ± 33.2	149.7 ± 55.2	161.5 ± 68.7	111.5 ± 32.8	114.5 ± 36.7	0.8596	0.9979	0.0467	0.1621
L_Lysine	169.4 ± 36.1	160.3 ± 28.8	202.1 ± 61.7	200.5 ± 48.4	176.8 ± 39.7	159.3 ± 36.6	0.8596	0.9979	0.0903	0.1133
L_Methionine	31.9 ± 7.8	28.5 ± 5.8	32.9 ± 10.1	33.3 ± 7.6	30.7 ± 9.2	27.6 ± 8.6	0.8769	0.9979	0.2812	0.2696
L_Phenylalanine	63.7 ± 12.2	57.3 ± 9.5	81.6 ± 21.2	76.9 ± 15.3	84.9 ± 41.7	68.4 ± 13.4	0.8596	0.9979	0.0015	0.4377
L_Threonine	164.7 ± 56.4	166.6 ± 39.5	142.8 ± 46.6	147. ± 38.1	134.2 ± 39.7	120.5 ± 30.1	0.7546	0.9979	0.0098	0.2607

^1^ Mean ± SD, ^2^ FDR-adjusted *p* values.

**Table 3:** Concentrations of acylcarnitines (µM) and TMAO at 5, 12 and 24 months between the meat and dairy groups ^1^.

							Meat Vs. Dairy Change over Time ^2^			
Acyl Carnitines	Meat 5 m	Dairy 5 m	Meat 12 m	Dairy 12 m	Meat 24 m	Dairy 24 m	12–5	24–12	12–5	24–12
TMAO	0.79± 0.99	1.46 ± 0.91	1.80 ± 1.70	1.42 ± 1.15	1.36 ± 1.18	1.21 ± 0.65	0.7546	0.9979	0.4147	0.8000
TMAO (meat only)									0.0431	0.2882
Acyl-C8:1	0.34 ± 0.14	0.31 ± 0.16	0.24 ± 0.16	0.33 ± 0.15	0.14 ± 0.08	0.13 ± 0.11	0.9064	0.9979	0.3122	0.0901
Acyl-C8	0.14 ± 0.06	0.12 ± 0.05	0.10 ± 0.06	0.12 ± 0.07	0.07 ± 0.05	0.07 ± 0.04	0.8596	0.9979	0.5238	0.2112
Acyl- C6	0.06 ± 0.02	0.05 ± 0.02	0.04 ± 0.02	0.05 ± 0.02	0.02 ± 0.01	0.02 ± 0.01	0.8596	0.9979	0.0903	0.0912
Acyl-C5:1	0.006 ± 0.003	0.006± 0.003	0.009± 0.005	0.012± 0.005	0.011± 0.003	0.008 ± 0.01	0.8596	0.9979	0.0040	0.5416
Acyl-C5	0.13 ± 0.06	0.11 ± 0.05	0.14 ± 0.10	0.16 ± 0.10	0.16 ± 0.09	0.11 ± 0.05	0.8596	0.9979	0.0136	0.1850
Acyl-C4	0.21 ± 0.11	0.22 ± 0.07	0.22 ± 0.11	0.24 ± 0.13	0.22 ± 0.06	0.15 ± 0.05	0.7546	0.8726	0.0467	0.0045
Aycl-C3	0.59 ± 0.18	0.61 ± 0.20	0.62 ± 0.26	0.68 ± 0.32	0.56 ± 0.17	0.46 ± 0.17	0.9990	0.9979	0.3122	0.1137
Acyl-C2	11.92 ± 4.06	12.92 ± 3.01	9.76 ± 5.76	11.41 ± 4.36	7.34 ± 2.06	7.29 ± 2.20	0.8596	0.9979	0.1427	0.0819
Acyl-C10:1	0.11 ± 0.06	0.12 ± 0.05	0.09 ± 0.06	0.13 ± 0.07	0.04 ± 0.03	0.06 ± 0.04	0.8596	0.9979	0.9111	0.0986
Acyl-C10	0.10 ± 0.09	0.12 ± 0.06	0.08 ± 0.08	0.11 ± 0.10	0.05 ± 0.06	0.06 ± 0.06	0.8596	0.9979	0.9111	0.3996
Acyl-C12	0.12 ± 0.13	0.15 ± 0.07	0.09 ± 0.08	0.14 ± 0.11	0.06 ± 0.07	0.09 ± 0.11	0.8596	0.9979	0.5238	0.7337
Acyl-C12:1	0.05 ± 0.07	0.07 ± 0.05	0.05 ± 0.06	0.07 ± 0.07	0.06 ± 0.06	0.08 ± 0.05	0.9723	0.9979	0.9111	0.8219

^1^ Mean ± SD, ^2^ FDR-adjusted *p* values. TMAO: trimethylamine N-oxide.

## Data Availability

The data presented in this study are available from the authors.
